# Habitudes toxiques et comportements alimentaires de 305 cas du cancer du sein colligés au centre Mohammed VI pour les traitements des cancers de Casablanca

**DOI:** 10.11604/pamj.2020.36.51.18869

**Published:** 2020-06-01

**Authors:** Drissi Houda, Imad Fatima Ezzahra, Bendahhou Karima, benider Abdelatif, Radallah Driss

**Affiliations:** 1 Laboratoire de Biologie et Santé, Unité de Recherche Associée CNRST, URAC-34, Faculté des Sciences, Ben M'sik Université Hassan II de Casablanca, B.P 5366 Maarif, Casablanca, Maroc; 2 Registre des Cancers de la Région du Grand Casablanca, Casablanca, Maroc; 3 Centre Mohammed VI pour le Traitement des Cancers, Casablanca, Maroc

**Keywords:** Cancer du sein, habitudes alimentaires, Maroc, habitudes toxiques, Breast cancer, diet, toxic habits, Morocco

## Abstract

**Introduction:**

Le cancer du sein représente un enjeu majeur de santé publique. L’objectif de ce travail est de décrire le profil sociodémographique, les habitudes toxiques et le profil alimentaire des patientes atteintes du cancer du sein traitées au centre Mohammed VI pour le traitement des cancers de Casablanca.

**Méthodes:**

Il s’agit d’une étude transversale portant sur 305 patientes atteintes de cancer du sein nouvellement diagnostiquées.

**Résultats:**

L’âge moyen des patientes est 50 ans. Cinquante-six pourcent (plus précisément 55,7%) de nos patientes sont mariées et 12,8% sont veuves. Parmi les femmes interrogées, 83% sont des femmes au foyer, 63,3% résident dans un milieu urbain et 47,9% sont analphabètes. Concernant les habitudes toxiques de nos patientes, seules 5,9% des femmes enquêtées ont déclaré avoir consommé de l’alcool et une minorité a déclaré être ex-fumeuse ou fumeuse, avec respectivement 4,3% et 2,3%. Quant au régime alimentaire, nos patientes consommaient le pain et la viande rouge avec une fréquence moyenne respectivement de 8,26 et 5,84 fois/semaine, et enfin les produits laitiers avec une fréquence moyenne de consommation allant de 3,55 à 4,57 fois/semaine. Par ailleurs, nos patientes consommaient plus de pomme de terre et des fruits frais, avec respectivement des moyennes de fréquence de 5,74 et 5,38 fois/semaine. Le thé reste également la boisson la plus consommée avec une moyenne de fréquence très élevée de 8,12 fois/semaine.

**Conclusion:**

À la lumière de nos résultats, la plupart des femmes interrogées, sont des femmes au foyer, analphabètes et résident dans un milieu urbain. Plusieurs aliments sont fréquemment consommés par nos patientes. En revanche, la consommation de substances psychoactives telles l’alcool et le tabac est faible. Des études analytiques type cas-témoins sont nécessaires afin d’établir d’éventuelles associations entre ces facteurs de risque et le cancer mammaire.

## Abstract (en)

**Introduction:**

Breast cancer is a major public health challenge. The purpose of this study is to describe the sociodemographic profile, toxic habits and diet profile of patients suffering from breast cancer treated in the Mohammed VI Cancer Treatment Center, in Casablanca.

**Methods:**

We conducted a cross-sectional study of 305 female patients newly diagnosed with breast cancer.

**Results:**

The average age of patients was 50 years; Fifty-six percent (55.7%) of patients were married and 12.8% were widows. Among the surveyed women, 83% were housewives, 63.3% lived in an urban environment and 47.9% were illiterate. With respect to patients’ toxic habits, only 5,9% of the surveyed women reported that they had consumed alcohol and a minority of them reported they were ex-smokers or smokers (4.3% and 2.3%respectively). With respect to diet, our patients consumed bread and red meat with an average frequency of 8.26 and 5.84 times/week respectively and, finally, dairy products with an average frequency ranging from 3.55 to 4.57 times/week. In addition, patients mostly consumed potatoes and fresh fruit, with an average frequency of 5.74 and 5.38 times/week respectively. Tea was the most widely consumed beverage, with a very high average frequency of 8.12 times/week.

**Conclusion:**

Given our results, most of the surveyed women were housewives, illiterate women and resided in an urban environment. Many foods were frequently consumed by our patients. On the other hand, the consumption of psychoactive substances such as alcohol and tobacco was low. Analytical case-control studies are necessary to establish a possible association between these risk factors and breast cancer.

## Introduction

Le cancer du sein constitue une préoccupation majeure de santé publique, il occupe chez la femme la première place en terme d’incidence dans le monde [1,2]. L’incidence standardisée mondiale était de 49,5 pour 100 000 femmes et au Maroc l’incidence standardisée sur la population était de 45,5 pour 100 000 femmes [3]. Selon l’OMS, il représente la principale cause de mortalité par cancer de la femme, avec plus de 450000 décès par an, soit 11% des décès par cancer [1,2]. L’objectif de cet article est de décrire le profil sociodémographique, les habitudes toxiques et le profil alimentaire des cas de cancer du sein chez une population féminine au niveau du centre Mohammed VI pour le traitement des cancers de Casablanca.

## Méthodes

Il s’agit d’une étude transversale portant sur 305 cas de cancer du sein nouvellement diagnostiqués et colligés au centre Mohammed VI de Casablanca pour le traitement des cancers. L’inclusion des patientes s’est faite de manière successive sur une période allant de janvier 2015 à décembre 2016. Les renseignements cliniques étaient notés dans un questionnaire standardisé administré en face à face des patientes et complétés à partir de dossiers médicaux des patientes. Les informations sociodémographiques comprenant l'âge au moment du diagnostic, l'état matrimonial, le niveau d’étude, le milieu de résidence et la situation professionnelle ont été également recueillies. Concernant le régime alimentaire et les habitudes toxiques (statut tabagique et consommation d’alcool), les questions portaient sur les habitudes des patientes une année avant le diagnostic du cancer. Différentes variétés d’aliments connues pour être des facteurs protecteurs (poissons, fibres alimentaires, produits laitiers) ou facteurs de risque du cancer du sein (viandes rouges, charcuteries, pâtes et céréales) ont été analysés. L’évaluation de la consommation de chaque aliment et chaque substance psychoactive est faite selon la moyenne de la fréquence de consommation hebdomadaire ± écart-type. L’analyse statistique des résultats a été réalisée par le logiciel R.

## Résultats

Durant la période d’étude, 305 patientes ont été prises en charge au sein du centre Mohammed VI pour les traitements des cancers. Les caractéristiques sociodémographiques analysées concernent l’âge, le statut marital, le niveau d’étude, la profession et le milieu de résidence (Tableau 1). L’âge moyen des patientes est 50,15 avec un écart type de 11,35 ans et des extrêmes allant de 24 à 95 ans. Plus de la moitié de nos patientes sont mariées avec une proportion de 55,7%, 17% sont célibataires et 12,8% sont veuves. Concernant le niveau d’étude, nos résultats ont montré que presque la moitié de la population étudiée (47,9%) sont des analphabètes, 23,3% ont un niveau d’étude primaire tandis que seules 19,3% ont un niveau d’étude secondaire. Le niveau universitaire ne représente que 2,6% de la population. Parmi les femmes interrogées, 83% sont des femmes au foyer, plus de 8% sont employées et seules 0,7% d’entre elles sont de hauts cadres, 1,3% parmi elles sont des étudiantes. Dans la population étudiée, 63,3% résident dans un milieu urbain et 28,2% résident dans un milieu rural. Les données concernant les habitudes toxiques de nos patientes sont représentées dans le Tableau 2. La majorité des patientes (93,4%) étaient non fumeuses, seules quelques patientes s’étaient déclarées ex-fumeuses ou fumeuses, avec respectivement 4,3% et 2,3%. Par ailleurs, seules 18 femmes enquêtées (5,9%) ont déclaré avoir consommé de l’alcool. Les habitudes alimentaires sont illustrées dans le Tableau 3 et Tableau 4; elles représentent la moyenne de fréquence de consommation des différentes aliments (végétaux, produits laitiers, céréales, viandes, boissons). Concernant la consommation des végétaux ; nos patientes consomment plus de la pomme de terre cuite et frite, avec respectivement une moyenne de fréquence de consommation hebdomadaire de 5,74 ± 3,23 fois/semaine et 3,17 ± 2,05 fois/semaine; la consommation des autres légumes est en moyenne de 4,20 ± 1,82 fois/semaine. Nos patientes consomment également des fruits frais, secs et oléagineux, avec respectivement une moyenne de fréquence de consommation hebdomadaire de 5,38 ± 2,71 fois/semaine; 3,25 ± 3,30 fois/semaine et 1,70 ± 2,13 fois/semaine. Par ailleurs, nous remarquons que les moyennes de fréquence de consommation hebdomadaire du lait, fromage et yaourt sont relativement proches, avec respectivement 4,57 ± 3,28 fois/semaine; 3,70 ± 3,64 fois/semaine et 3,55 ± 2,62 fois/semaine. Les patientes prennent également beaucoup plus du pain que les pâtes et le riz avec une moyenne de fréquence de consommation hebdomadaire respectivement de 8,26 ± 2,42 fois/semaine ; 3,91 ± 1,64 fois/semaine et 3,47 ± 1,87 fois/semaine. Nos résultats ont révélé aussi que les patientes consomment plus fréquemment la viande rouge suivi par la volaille et le poisson, avec une moyenne de fréquence de consommation hebdomadaire respectivement de 5,84 ± 2,63 fois/semaine; 5,10 ± 2,30 fois/semaine et 3,71 ± 1,75 fois/semaine. Parallèlement, nos patientes consomment moins de charcuteries (2,44 ± 2,67fois/semaine), mais plus d’œufs, avec une fréquence moyenne de consommation hebdomadaire de 4,40 ± 2,57 fois/semaine. Concernant les boissons, nos résultats ont montré que la moyenne de fréquence de consommation hebdomadaire du thé est plus élevée que celles du jus de fruit, café et soda, avec respectivement 8,12 ± 3,07 fois/semaine ; 3,83 ± 2,35 fois/semaine; 3,10 ± 3,5 fois/semaine et 2,71 ± 2,89 fois/semaine.

**Tableau 1:** Description générale de la population étudiée

Caractéristiques	Effectif	Nombre de cas (%)
**Age moyen (ans)**	305	50,1
**Statut marital**		
Célibataire	52	17
Mariée	170	55,7
Divorcee	44	14,4
Veuve	39	12,8
**Niveau d’étude**		
Analphabèe	146	47,9
Ecole coranique	21	6,9
Primaire	71	23,3
Secondaire	59	19,3
Universitaire	8	2,6
**Profession**		
Femme de foyer	253	83
Employée	26	8,5
Fonctionnaire	19	6,2
Haut cadre	2	0,7
Retraitée	1	0,3
Autre: (étudiante)	4	1,3
**Milieu de résidence**		
Urbain	193	63,3
Suburbain	26	8,5
Rural	86	28,2

**Tableau 2:** Les habitudes toxiques chez les patientes

Caractéristiques	Effectif	Nombre de cas (%)
**Tabagisme**		
Fumeuse	7	2,3
Non fumeuse	285	93,4
Ex- fumeuse	13	4,3
**Consommation d’alcool**		
Oui	18	5,9
Non	287	94,1

**Tableau 3:** La moyenne de la fréquence de consommation hebdomadaire des certains aliments chez les patientes atteintes de cancer du sein

Aliments	Consommation moyenne±écart type
**Végétaux**	
Frites	3,17±2,05
Pomme de terre	5,74±3,23
Légumes	4,20±1,82
Légumes secs	3,18±1,81
Fruit	5,38±2,71
Fruit oléagineux	1,70±2,13
Fruit secs	3,25±3,30
**Produits laitiers**	
Lait	4,57±3,28
Yaourt	3,55±2,62
Fromage	3,70±3,64
**Céréales**	
Pâtes	3,91±1,64
Riz	3,47±1,87
Pain	8,26±2,42
**Viandes et œufs**	
Viande rouge	5,84±2,63
Volailles	5,10±2,30
Charcuterie	2,44±2,67
Poisson	3,71±1,75
œuf	4,40±2,57
**Boissons**	
Soda	2,71±2,89
Jus de fruit	3,83±2,35
Café	3,10±3,5
Thé	8,12±3,07

**Tableau 4:** La fréquence de consommation des certains aliments chez les patientes atteintes du cancer du sein

Aliment	Jamais	1-3/mois (4 semaines)	1-3/sem	4-7/sem
	Nombre de cas (%)	Nombre de cas (%)	Nombre de cas (%)	Nombre de cas (%)
**Légumes**	106(34,8)	2 (0,7)	66 (21,6)	131 (43)
**Légumes secs**	22 (7,2)	128 (42)	148 (48,5)	7 (2,3)
**Fruits**	116 (38)	57 (18,7)	109 (35,7)	23 (7,5)
**Viande rouge**	10 (3,3)	34 (11,1)	143 (46,9)	118 (38,7)
**Volailles**	12 (3,9)	56 (18,4)	167 (54,8)	70 (23)
**Charcuterie**	138 (45,2)	38 (12,5)	115 (37,7)	14 (4,6)
**Poisson**	11 (3,6)	108 (35,4)	173 (56,7)	13 (4,3)

## Discussion

Le cancer du sein représente le premier cancer de la femme et la première cause de mortalité féminine [1,2]. Selon les données des registres de Rabat et du grand Casablanca [3,4], l’incidence du cancer du sein au Maroc est relativement plus élevée que dans les autres pays du Maghreb, mais elle reste nettement inférieure aux incidences retrouvées dans les pays occidentaux, où les taux d’incidence sont supérieurs à 80 pour 100000 personnes [5]. Beaucoup de facteurs complexes sous-tendent cette augmentation d’incidence, notamment la structure des populations (telle que l’âge, statut marital, et milieu de résidence), le style de vie, l'environnement, le statut socioéconomique, le régime alimentaire.

Le premier volet de notre étude s’oriente vers la description et l’estimation de la prévalence des facteurs de risque qui pourraient prédisposer la femme au cancer mammaire, l’âge est parmi ces facteurs de risque les plus importants vis-à-vis du cancer du sein. Dans notre étude, nous retenons que l’âge moyen de nos patientes est 50,15±11,35 ans et des extrêmes allant de 24 à 95 ans. Nos résultats concordent avec ceux du registre des cancers de la région du Grand Casablanca [3]. Comme en France, la maladie est rare chez les femmes de moins de 30 ans [6] et le risque augmente entre 50 et 75 ans, avec près des deux tiers [7]. Plusieurs études suggèrent que le cancer du sein est plus agressif chez les femmes âgées de 40 à 49 ans [8]. Une étude prospective menée en Islande entre 1982 et 2004, sur une population âgée de 20 à 64 ans, a conclu à une association positive entre le risque de cancer du sein et le niveau d’étude [9]. Concernant nos patientes, 47,9% sont analphabètes. Ces résultats sont comparables à ceux rapportés dans la littérature [10-12] et soulignent les difficultés d’accès à l’information pour nos patientes et leur méconnaissance des facteurs de risque et les symptômes liés au cancer du sein.

En outre, concernant les habitudes toxiques de nos patientes, la majorité d’entre-elles (93,4%) sont non fumeuses, 4,3% et 2,3% sont des ex-fumeuses et des fumeuses, respectivement. Ainsi, il apparait chez notre population d’étude, que la consommation du tabac n’est pas très fréquente et ne semble pas être associée au risque de développer le cancer du sein. Cependant, d’autres études internationales démontrent l’existence d’une association évidente entre la consommation du tabac et le risque excessif du cancer du sein, d’autant plus que le tabagisme est initié précocement et avant la première grossesse [13]. Par ailleurs, dans la population étudiée, seules 18 femmes enquêtées (5,9%) ont signalé qu’elles consommaient l’alcool. Cependant, dans d’autres études, le lien entre la consommation d’alcool et le cancer du sein est bien établi. McCarty *et al*. rapportaient une élévation du risque relatif du cancer mammaire chez les consommatrices d’alcool, que ce soit avant ou après la ménopause [14]. Le second volet de cette étude concerne l’exploration des habitudes alimentaires chez les patientes, dans l’étiologie du cancer mammaire. En référence aux facteurs de risque cités dans la littérature, plusieurs études menées à travers le monde ont abouti à l’implication des facteurs nutritionnels [15-20].

Dans notre étude, la consommation des viandes rouges est élevée. Presque 40% des femmes interrogées en mangent quotidiennement. Ce résultat est en accord avec plusieurs méta-analyses et études de cohorte [16-18] et souligne que la consommation de viande rouge, essentiellement composée de graisse animale, de fer hémique et de cancérogènes chimiques pouvant s'accumuler pendant la cuisson et/ou la transformation, était associée à un risque accru de cancer du sein. Une attention particulière est portée à la consommation de la viande blanche (volaille et poissons). Plus de 50% des femmes enquêtées ont déclaré consommer le poisson et la volaille de façon régulière. Ce résultat est en discordance avec de nombreux travaux ayant montré une diminution de risque de cancer mammaire avec une consommation élevée de viande blanche [17]. Il est à noter que l’effet protecteur de la consommation des viandes blanches vis-à-vis des tumeurs reste inexpliqué [17]. Nous constatons également que la moyenne de la fréquence de consommation hebdomadaire des fruits et des légumes par les femmes enquêtées est importante, avec respectivement (5,38 ± 2,71) et (4,20 ± 1,82). L’équipe de Taylor & Francis-group avait souligné une relation inverse entre une consommation régulière des fruits et légumes et le risque de carcinogenèse, ce qui apparemment n’est pas le cas chez notre population d’étude [16]. Cependant, 38% de nos patientes n’ont jamais mangé de fruits et 34,8% n’ont jamais consommé de légumes. Ces taux peuvent être expliqués par l’analphabétisme et le bas niveau socio-économique des femmes suivies dans le centre. Pour d’autres, il s’agit d’un véritable changement dans le style de vie, le passage d'une alimentation à base de céréales, des légumes et des fruits à une alimentation riche en viandes, et une consommation de plus en plus fréquente des «fastfood» et des produits industrialisés. Ces transformations dans notre mode vie font craindre une augmentation de l'incidence des cancers du sein dans notre pays.

Les résultats de notre enquête ont montré également que la moyenne de consommation du café et de3,10 avec un écart type de 3,5, alors qu’un effet bénéfique a été évoqué pour les grandes consommatrices [18]. Concernant l’apport en produits laitiers, la consommation moyenne est presque de 5 prises par semaine. Les résultats de nombreuses recherches confirment que les femmes ayant un grand apport en produits laitiers ont un risque réduit de cancer du sein. Il a été rapporté une diminution du risque chez les plus fortes consommatrices de produits laitiers et de calcium, essentiellement chez les femmes avant la ménopause [19,20].

## Conclusion

Le cancer du sein est une pathologie cliniquement hétérogène et multifactorielle, sa prise en charge est complexe et l’évaluation des facteurs de risque est une étape importante pour des mesures de prévention efficaces. Le rôle de l’alimentation dans le risque de cancer du sein dans les pays en voie du développement a été le moins étudié. De plus, la majorité des études s’est concentré sur l’alimentation pendant la vie adulte. Le sein peut être l’organe le plus sensible aux effets des habitudes alimentaires au début de la vie, en particulier avant la puberté. A la lumière de nos résultats, notre série d’analyses donne une vue globale sur l’alimentation, les habitudes toxiques et le profil sociodémographique chez nos patientes. Ces résultats doivent être complétés par d’autres études analytiques afin d’établir l’association entre ces facteurs et le cancer du sein.

### Etat des connaissances actuelles sur le sujet

- Le cancer du sein constitue une préoccupation majeure de santé publique;
- Le cancer mammaire est le premier cancer féminin responsable d’une mortalité élevée à travers le monde;
- Plusieurs études épidémiologiques et expérimentales menées à travers le monde ont souligné l’implication du mode de vie, incluant les facteurs nutritionnels, dans la survenue de cancer du sein.

### Contribution de notre étude à la connaissance

- Cette étude relate les comportements toxique et alimentaire, une année avant le diagnostic du cancer du sein, chez des patientes majoritairement analphabètes et de bas niveau socioéconomique;
- La consommation très faible du tabac et l’alcool notée chez les patientes ne semble pas être associée au risque de développer le cancer du sein mais continuent d’être considérées comme des facteurs aggravants et évitables de la maladie;
- Il ressort de notre étude que certains aliments sont susceptibles d'intervenir dans le développement du cancer du sein, notamment la viande rouge, les féculents, les produits laitiers et le thé (boisson généralement sucrée).

## Conflits d’intérêts

Les auteurs ne déclarent aucun conflit d’intérêts.
